# Construction, expression, and in vitro assembly of virus-like particles of L1 protein of human papillomavirus type 52 in *Escherichia coli* BL21 DE3

**DOI:** 10.1186/s43141-021-00281-5

**Published:** 2022-02-07

**Authors:** Apon Zaenal Mustopa, Lita Meilina, Shasmita Irawan, Nurlaili Ekawati, Alfi Taufik Fathurahman, Lita Triratna, Arizah Kusumawati, Anika Prastyowati, Maritsa Nurfatwa, Ai Hertati, Rikno Harmoko

**Affiliations:** Research Center for Biotechnology, National Research and Innovation Agency (BRIN), Bogor, 16911 Indonesia

**Keywords:** *E. coli* BL21 DE3, Human papillomavirus (HPV), pETSUMO, L1 protein

## Abstract

**Background:**

A major discovery in human etiology recognized that cervical cancer is a consequence of an infection caused by some mucosatropic types of human papillomavirus (HPV). Since L1 protein of HPV is able to induce the formation of neutralizing antibodies, it becomes a protein target to develop HPV vaccines. Therefore, this study aims to obtain and analyze the expression of HPV subunit recombinant protein, namely L1 HPV 52 in *E. coli* BL21 DE3. The raw material used was L1 HPV 52 protein, while the synthetic gene, which is measured at 1473 bp in pD451-MR plasmid, was codon-optimized (ATUM) and successfully integrated into 5643 base pairs (bps) of pETSUMO. Bioinformatic studies were also conducted to analyze B cell epitope, T cell epitope, and immunogenicity prediction for L1HPV52 protein.

**Results:**

The pETSUMO-L1HPV52 construct was successfully obtained in a correct ligation size when it was cut with *EcoR*I. Digestion by *Eco*RI revealed a size of 5953 and 1160 bps for both TA cloning petSUMO vector and gene of interest, respectively. Furthermore, the right direction of construct pETSUMO-L1HPV52 was proven by PCR techniques using specific primer pairs then followed by sequencing, which shows 147 base pairs. Characterization of L1 HPV 52 by SDS-PAGE analysis confirms the presence of a protein band at a size of ~55 kDa with 6.12 mg/L of total protein concentration. Observation under by transmission electron microscope demonstrates the formation of VLP-L1 at a size between 30 and 40 nm in assembly buffer under the condition of pH 5.4. Based on bioinformatics studies, we found that there are three B cell epitopes (GFPDTSFYNPET, DYLQMASEPY, KEKFSADLDQFP) and four T cell epitopes (YLQMASEPY, PYGDSLFFF, DSLFFFLRR, MFVRHFFNR). Moreover, an immunogenicity study shows that among all the T cell epitopes, the one that has the highest affinity value is DSLFFFLRR for Indonesian HLAs.

**Conclusion:**

Regarding the achievement on successful formation of L1 HPV52-VLPs, followed by some possibilities found from bioinformatics studies, this study suggests promising results for future development of L1 HPV type 52 vaccine in Indonesia.

## Background

Human papillomavirus (HPV) causes the most common viral infection in the human reproductive tract system; it consists of small double-stranded DNA pathogens that infect the epithelium. Recently, more than 200 HPVs have been identified and differentiated by their genomic sequences. Furthermore, around 40 types of those HPV’s variants infect mucosal epithelium, and epidemiology studies found its strong association with cervical cancer. The HPV type-16 causes approximately 50% of cervical cancers globally, while its combination with type-18 increases the cases up to 66%. Additionally, the five-high risk types (31, 33, 45, 52, and 58) are responsible for 15% cervical cancers, and 11% of all cases associated with HPV infection [1]. In Indonesia, the highest HPV prevalence is caused by HPV 52 (23.2%), 16 (18%), 18 (16.1%), and 39 (11.8%). The HPV 52 is known to be the most prevalent type in Indonesia, while another study also identified it to be the frequent cause of cervical cancer in East Asian Countries (Japan, South Korea, Taiwan, and China) than other parts of the world [2]. Therefore, based on these reports, HPV 52 prophylactic vaccine is suggested to be introduced in Indonesia, in order to curtail the prevailing human papillomavirus.

Human papillomavirus consists of six genes located in the early region of the genome (*E1*, *E2*, *E4*, *E5*, *E6*, *E7*) and two (*L1* and *L2*) in the late region. The exterior surface of papillomavirus virion is composed of a pentameric L1 capsomer and accommodates up to 72 molecules of a minor capsid protein L2, which is only minimally exposed. These dominant L1 features obviously mediate the initial attachment to the host cells. Additionally, the recombinant L1 protein spontaneously self-assembled into a highly immunogenic structure, that closely mimics the original surface of the HPV without genetic material [3]. Two prophylactic HPV vaccines have been licensed since 2006/2007 and composed virus-like particles (VLPs) of the L1 capsid protein. These include Cervarix®, a bivalent HPV (bHPV) 16/18, and Gardasil® (also known as Silgard), a quadrivalent type (qHPV) 6, 11, 16, 18 [3]. A 9-valent recombinant protein subunit HPV vaccine (9vHPV, Gardasil 9) has also been licensed for use, which prevents types 6, 11, 16, 18, 31, 33, 45, 52, and 58.

The Indonesian government has set a national HPV vaccination program; however, the main obstacle encountered during implementation includes the inability to produce the vaccine domestically and the need to import from other countries. Besides the concern in regards of vaccine supplies, it also requires a huge budget in the purchase. Therefore, a solution regarding the availability of the vaccines should be provided, by implementing domestic production. This study focused on the production of VLP L1 protein that is highly recommended as a future vaccine to overcome for HPV type 52 infection in Indonesia. The VLP is obtained through L1 production in various expression systems, including mammalian cells, plants, bacteria, insects, and yeast. Although eukaryotic cells produce highly effective vaccines, this vector has several drawbacks, including high production and purification costs. In contrast, the bacterial expression system (*Escherichia coli*) has already been used in heterologous recombinant proteins with many advantages, such as faster growth, low production cost, ease during gene manipulation, and scaling up [4]. The bacterial expression system does not directly produce VLPs; it is achieved after a purification process and assembled into VLPs [5, 6]. To obtain the highest protein yield, an appropriate expression vector should be validated to give an efficient method for high-level gene expression.

Formation of inclusion bodies in bacterial hosts poses a major challenge for large-scale production [6]. The SUMO fusion protein is a small ubiquitin-related modifier that enhances the solubility of the expressed recombinant protein [7]. This study used a codon-optimized L1 HPV 52 to induce higher expression in *E. coli* BL21 DE3 that is composed of a Champion pET SUMO protein expression system. This system facilitates an easy purification process for obtaining the native L1 protein.

## Methods

### Bacterial strains and plasmids

The bacterial strains and plasmids used in this study are shown in Table 1.

**Table 1:** Bacterial strains and plasmids used in this study

Strains and plasmids	Characteristics	Source
*E. coli* Mach1™ -T1^R^	Cloning host strain	Invitrogen, USA
*E. coli* BL21 DE3	Expression host strain	Invitrogen, USA
pD451-MR_L1-HPV52	Kan^r^; T7 Promoter, cloning vector	ATUM, USA
pETSUMO	Kan^r^; T7 promoter; expression vector	Invitrogen, USA

*Kan*^*r*^, kanamycin resistance

### Construction of truncated L1 HPV type 52 in *E. coli* BL21 DE3

The complete genome used as template for HPV 52 L1 protein sequence was obtained by the genome analysis from the database of the National Centre for Biotechnology Information (NCBI) (https://www.ncbi.nlm.nih.gov/). All the sequences were then subjected to multiple alignment analysis, and the HPV 52 L1 protein sequence having 100% similarity was selected as the template (GenBank accession no. APQ44871.1) [8]. The synthetic gene of L1-HPV52 used was designed as truncated L1 for its 26 amino acids in the N-terminal and the codon-optimized by ATUM Company DNA 2.0 Gene Design & Synthesis (Newark, California). It was further integrated into pD451-MR inducible plasmid (pD451-MR: 399524) and transformed into *E. coli* BL21 DE3 expression vector. The isolation of L1-HPV52 synthetic gene from plasmid pD451 was carried out by polymerase chain reaction (PCR) (Axygen), using forward 5′-ATGCCAGTCCCTGTTTCTAAAG- ′3 and reverse 5′ -TCATTAACGCTTAACTTTTT-′3 primers. The PCR conditions used were pre-denaturation (94 °C for 2 min), denaturation (98 °C for 10 s), annealing (53 °C for 30 s), extension (68 °C for 30 s), and post-extension (72 °C for 8 min) (Manual Instruction KOD FX Neo). The amplicon was confirmed using 1% agarose gel and visualized in a UV Transilluminator. Isolated L1-HPV52 genes were further ligated into pETSUMO expression plasmid vector. A total of 9 μl of PCR products and 1 ul of 10X A-attachment mix (Toyobo) were incubated at 60 °C for 30 min and ligated into pETSUMO (25 μg) using T4 DNA Ligase (NEB) for overnight incubation at 4 °C prior to transformation into *E. coli* Mach1™ -T1^R^ cloning host by heat shock method. Confirmation of putative transformants was performed by colony PCR and plasmid digestion using *EcoRI* enzyme (NEB), which perform double digestion on the template gene and *XbaI* (NEB), which produce linear gene product. The pETSUMO-L1HPV52 (+) construct was transformed into expression vector host *E. coli* BL21 (DE3), followed by DNA sequencing using SUMO forward (5′-AGATTCTTGTACGACGGTATTAG-3′) and T7 Reverse (5′-TAGTTATTGCTCAGCGGTGG-3′) primers set at the 1st Base Laboratories (Malaysia) as validation of successful transformation into expression host. The sequence analysis was performed with the BLAST method (https://blast.ncbi. nlm.nih.gov/Blast).

### Expression and purification of recombinant His-SUMO-L1-HPV52 protein

The recombinant *E. coli* BL21 DE3 bacteria harboring pETSUMO-L1HPV52 were grown on liquid Luria Bertani (LB) medium with an addition of 100 μg/mL of kanamycin. Then, 1% of pre-cultured bacteria was inoculated into a fresh LB medium and incubated at 37 °C for ± 3 h, until OD_600_ reached ~0.5–0.6, which is a logarithmic phase of the *E. coli* BL21 DE3. The culture induction used 0.5 mM IPTG along with an addition of 1% (v/v) glucose and incubated at 20 °C for ± 5 h, until the OD_600_ reached ~1.0 as the stationary phase of the *E. coli* BL21 DE3 [7, 9]. The bacterial cells were harvested by centrifugation at 6000 rpm for 10 min. The cell pellets were then resuspended with 0.5% lysis buffer, containing 50 mM potassium phosphate pH 7.8, 400 mM NaCl, 100 mM KCl, 10% glycerol, 0.1% triton X-100, 5 mM imidazole, 100 μg/mL lysozyme, and 1 mM PMSF. The protein was harvested by centrifugation at 12,000 rpm for 10 min. The supernatant was collected as crude protein samples and stored at – 20 °C for further analysis. The purification of L1-HPV52 protein with His-6x and the SUMO protein fusion were carried out using the Ni-NTA agarose (Thermo) procedure in native conditions [10].

### SUMO cleavage and assembly of L1-HPV52

Further characterization was performed to obtain purified L1-HPV52. The protein fusion tag should be cleaved on the SUMO cleavage site using SUMO protease. As much as 10 units of SUMO protease was used to cleave SUMO fusion tag from the recombinant His-SUMO-L1 HPV52 and processed in 10x protease buffer without NaCl and 1x Native Binding Buffer (- salt) of the total volume 1.5 mL, in the rotating resin overnight (±16 h) at 4°C. The supernatant was collected per 0.5 mL sample, and purified L1-HPV52 (100 μg/mL) was assembled with buffer pH 5.4 (1M NaCl, 40 mM sodium acetate) for 30 min at 25°C [9]. Observations of self-assembled L1-HPV52 VLP were carried out using a transmission electron microscope (TEM-JEOL JEM 1400).

### Protein characterization

Characterization of purified His-SUMO-L1HPV52 protein was analyzed using SDS-PAGE 10% [34] and Western blot at a voltage of 110 V for ± 90 min [11]. The total protein concentration was determined using Bicinchoninic Acid (BCA) assay kit (Thermo Fisher Scientific, USA) [12].

### Bioinformatic studies, B cell epitope prediction, T cell epitope prediction, and immunogenicity analysis

The L1HPV52 sequencing results both from pDH451-MR_L1HPV52 and pETSUMO_L1HPV52 were checked and translated using BioEdit ver 7.2 and ExPASy DNA translate tool. Subsequently, a comparison was performed between DNA and amino acid (aa) sequence with Basic Local Alignment Search Tools (BLAST) ((https://www.ncbi.nlm.nih.gov/)).

Epitope B cell prediction was done using IEDB analysis (http://tools.iedb.org/ellipro/). The L1HPV52 aa sequence as the translation result from ExPASy in linear form was used to predict B cell epitope. Prediction of the position of B cell epitopes in monomer, pentamer, and VLP of L1HPV52 was done in Swiss-Pdb Viewer (SPDBv) v4.1.

The immunogenicity server (http://tools.immuneepitope.org/immunogenecity/) was used for Epitope T cell and immunogenicity prediction. In this study, we used some Indonesian HLAs, classes I and II. Moreover, Swiss-Pdb Viewer (SPDBv) v4.1 was used to predict the position of T-cell epitopes in monomer, pentamer, and VLP of L1HPV52 forms.

## Results

### Construction of recombinant pETSUMO-L1HPV52 in *E. coli* BL21 DE3

**Fig. 1:**
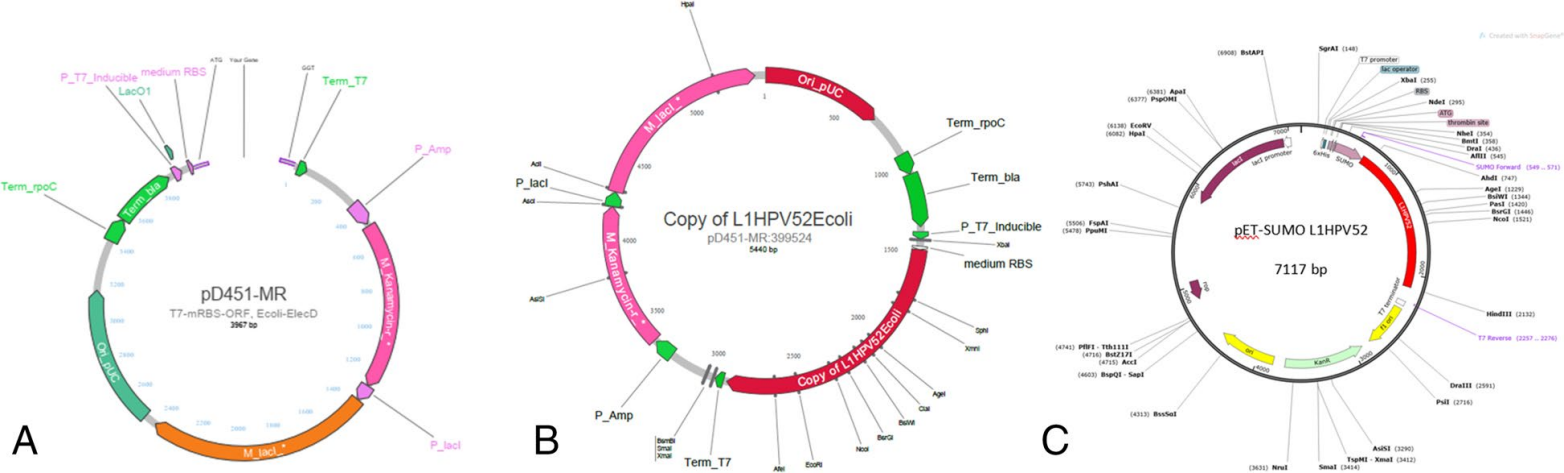
Arrangements of L1-HPV52 gene target inside both cloning and expression vectors. **A** Plasmid map of original pD451-MR from ATUM, which has 3967 bps. **B** The plasmid pD451-MR harboring codon-optimized truncated L1-HPV52 with 26 aa truncations, which makes 5440 bps. **C** Integration of L1-HPV52 into pETSUMO-L1HPV52 expression vector created a total length of 7116 bps

**Fig. 2:**
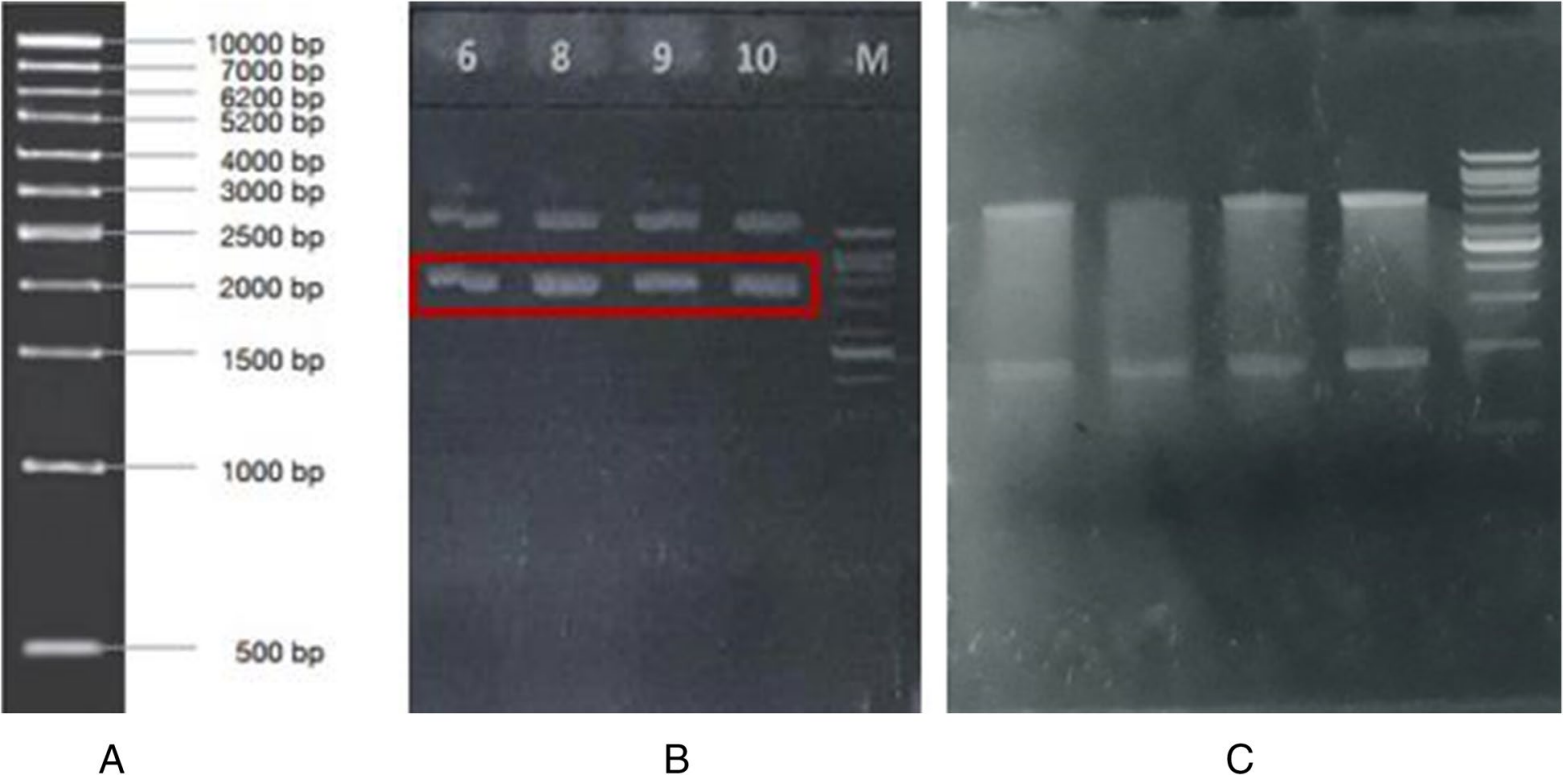
Electrophoregram of plasmid isolation and digestion as a part of transformants selection step in *E. coli* Mach1™ -T1^R^ harboring pD451-MR_L1-HPV52. The DNA bands were compared to 1kb DNA ladder and code numbers 6, 8, 9, 10 represent colonies number 6, 8, 9, and 10. M: DNA Ladder 1kb (Vivantis); **A**: size of 1 kb DNA marker; **B**: uncut plasmid, which demonstrates 2 types of DNA bands, nicked and supercoiled; **C**: plasmid digestion using 2 digestion enzymes, *Eco*RI and *Xba*I, presentation of 2 DNA bands are well defined at 4507 and 933 bps

**Fig. 3:**
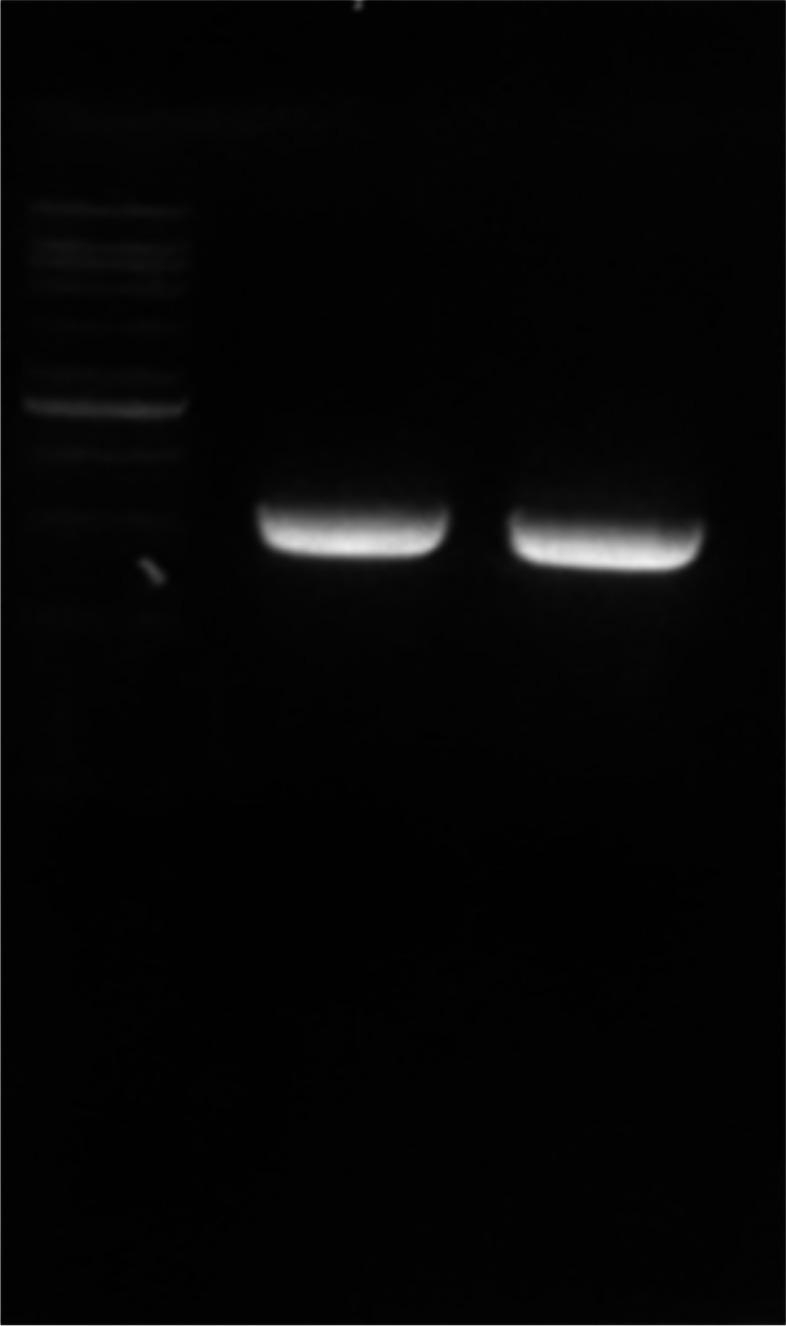
Electrophoregram of positive transformant Colony number 9 harboring L1-HPV52, amplification was done using a specific primer with annealing temperature of 53°C resulting in clear DNA bands at 1473 bps. M: 1 kb DNA Ladder (Vivantis)

The recombinant pD451-MR_L1-HPV52 synthetic gene (ATUM) consists of 5440 bps pD451-MR cloning vector carrying 1473 bps of L1-HPV52 target gene (Fig. 1B). Plasmid sequencing of the cloning host harboring pD451-MR_L1-HPV52 (colony 9 and 10) was done to select positive transformants (data not shown). Along with the plasmid-sequencing step, isolation of pD451-MR_L1-HPV52 plasmid was also carried out to confirm the insertion in pD451-MR plasmid (Fig. 2B, C). Figure 2B is the uncut pD451-MR_L1-HPV52 plasmid. There are 2 plasmid bands that are not in their proper position because the plasmid is in circular shape. Thus, it can form nicked and supercoiled states, which cause different migration speeds. Plasmids in supercoiled form will migrate faster than those in nicked form in agarose gel [25]. The pD451-MR_L1-HPV52 was digested with *Eco*RI and *Xba*I (Fig. 2C). The *Eco*RI can cut pD451-MR_L1-HPV52 at position 2543 while *Xba*I can cut this plasmid at position 1411. As the result, there are 2 bands in a size of 4507 bp and 933 bp. All the results mentioned above are useful to confirm specific primer design to obtain the L1-HPV52. Furthermore, the insert gene itself was amplified using a specific primer at 53°C and generated a single band under 1500 bp DNA ladder. The PCR product was confirmed as L1-HPV52 size at 1473 bps (Fig. 3). These results confirmed that the L1-HPV52 gene was integrated in the right size and direction.

**Fig. 4:**
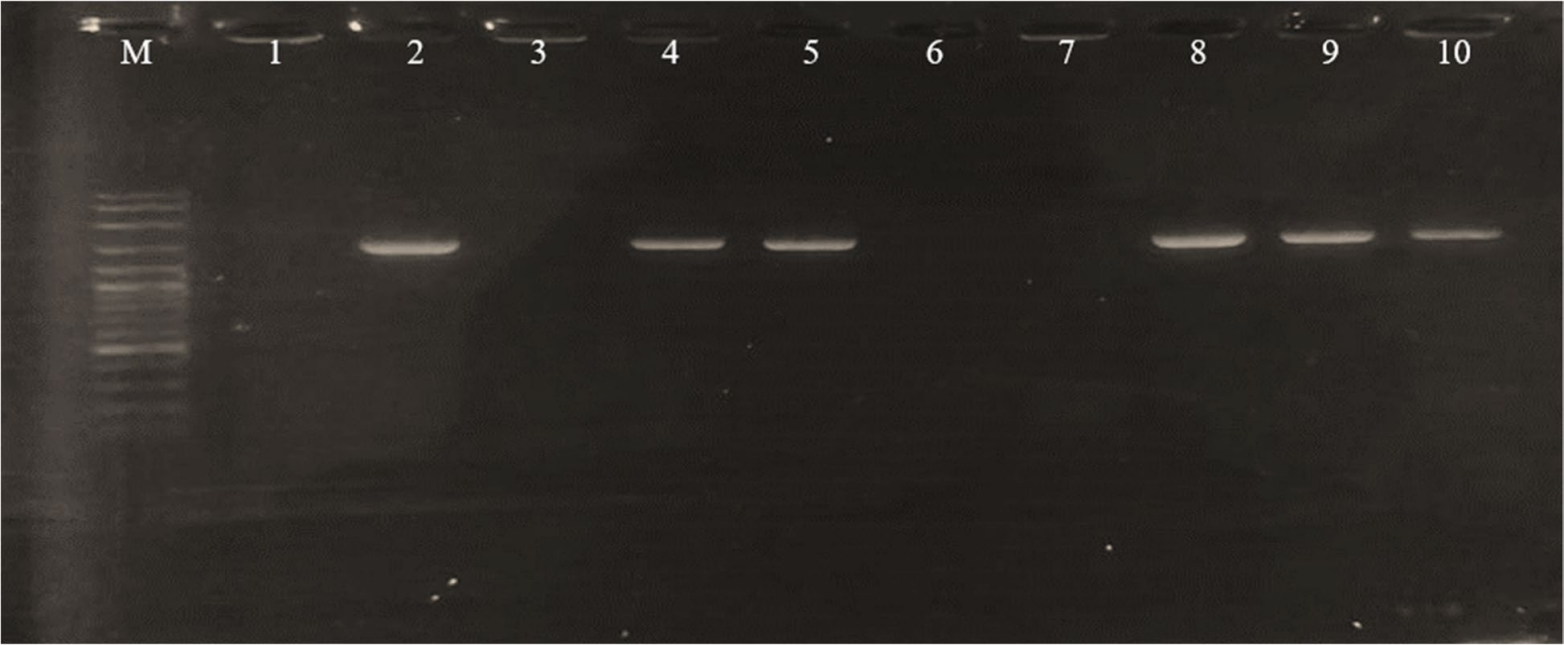
Electrophoregram of PCR colonies results to confirm the presence of pETSUMOL1HPV52 inside *E. coli* BL21 DE3 expression host**.** M: 100 bp DNA ladder (Vivantis); code number 1–10 represents transformant colonies number. As seen on the picture, 6 samples out of 10 confirmed of having the L1HPV52

**Fig. 5:**
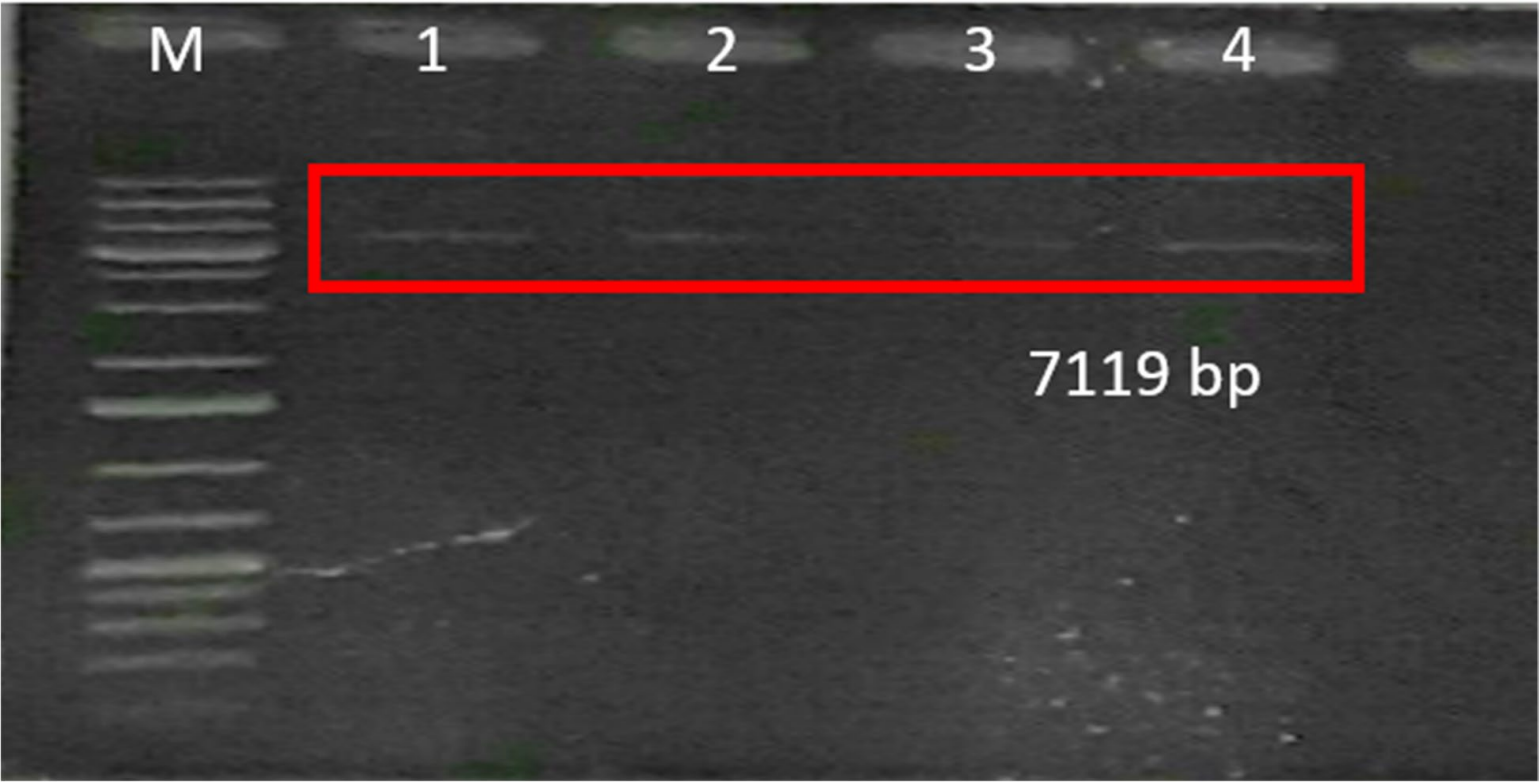
Electrophoregram of plasmid pETSUMO-L1HPV52 extracted from *E. coli* BL21 DE3 expression vector before production. M: O’Gene Ruler 1kb plus DNA ladder (Thermo); code number 1–4 represent transformant’s colonies number. Only 1 colony out of 6 colonies was having pETSUMO-L1HPV52 plasmid in right direction. After colony multiplication, the 4 of those multiplied colonies were re-tested by digestion using *XbaI* and DNA band at 7113bp were seen on agarose gel as positive confirmation of integration into expression vector

The recombinant transformation of pETSUMO-L1HPV52 into *E. coli* BL21 DE3 was carried out using a heat shock approach [13], and confirmed by colony PCR. The results show that there were six colonies (2, 4, 5, 8, 9, and 10) confirmed as positive, carrying the pETSUMO-L1HPV52 construct with band sizes 1473 bp (Fig. 4). Further confirmation of positive colonies was carried out by digesting the pETSUMO-L1HPV52 to identify the presence of ligated inserts and determine the direction in the TA cloning-pETSUMO vector. The plasmids of the confirmed colony were digested using *XbaI* (NEB), which only cut the plasmid in one site and generated linearized DNA. From the results shown, only one colony (number 9) was confirmed to carry the insert in the right direction, by demonstrating a DNA band on the size of 7113 bp after further digestion using *XbaI* restriction enzyme (Fig. 5).

### Expression, purification, and characterization of recombinant L1-HPV52 protein

**Fig. 6:**
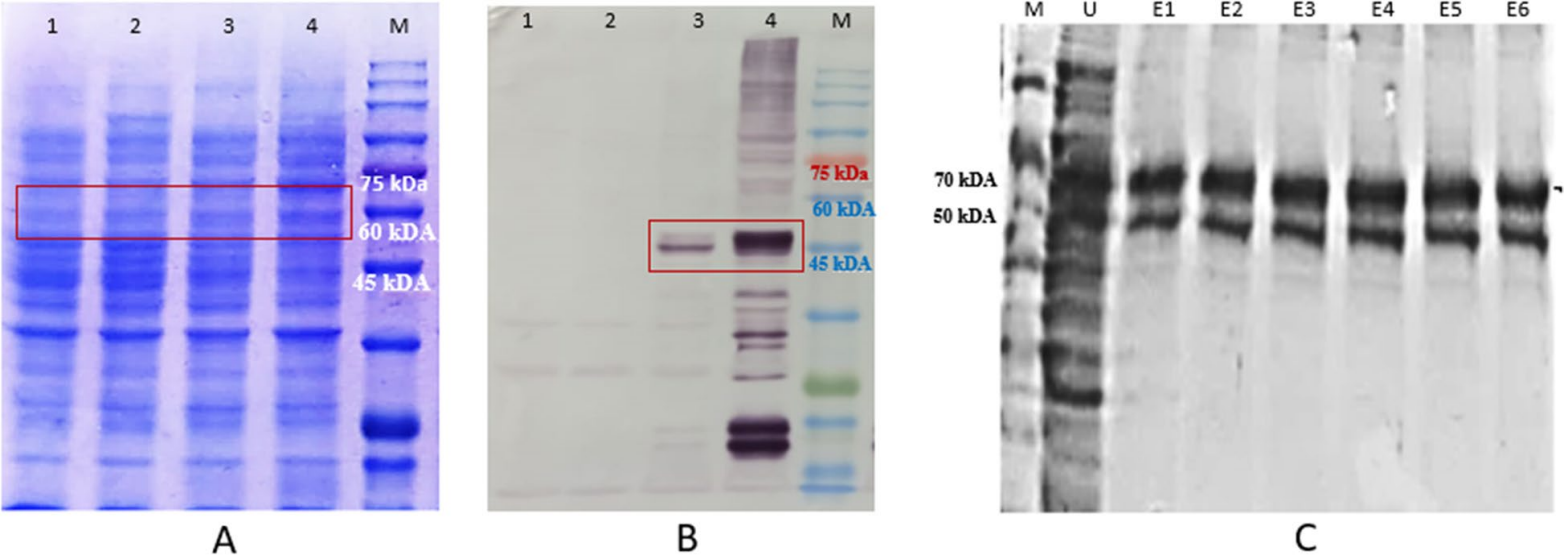
Analysis of L1-HPV52 protein production in shake flask cultivation using **A** SDS-PAGE of crude samples and **B** western blot with anti-His-AP. Both Fig. 6A and B share the same protein arrangement; lane 1: BL21 DE3 no plasmid and uninduced; lane 2: BL21 (DE3 no plasmid and induced with 0.3 mM IPTG; lane 3: BL21 DE3 harboring pETSUMO-L1HPV52 and uninduced; lane 4: BL21 DE3 harboring pETSUMO-L1HPV52 and induced with 0.3 mM IPTG; lane M molecular weight size marker (kDa). **C** SDS-PAGE profile of purified L1-HPV52 with a fusion tag

Protein characterization was performed by SDS-PAGE analysis, to confirm the size of the recombinant protein constructed in pETSUMO-L1HPV52 expression vector and expressed in *E. coli BL21* DE3 expression host. Figure 6 shows bioinformatic analysis of 3-dimensional structure of His-SUMO-L1HPV52 and L1-HPV52 proteins. To determine whether the protein was properly expressed, SDS-PAGE analysis in Fig. 6A shows a protein band at ~68 kDa, which made a different profile between induced and uninduced samples. These results were also confirmed by immunoblotting, that the recombinant bacterial harboring the targeted gene was detected (Fig. 6B). The purified His-SUMO-L1HPV52 profile generated 2 bands, which were suspected to be lacking in the resin washing process (Fig. 6C). Therefore, other protein bands were still visible, both in the crude and eluate samples. The total protein obtained in every purification step was measured using BCA assay (Table 2). The concentration obtained in Ni-NTA purification was low, because presumably, there were still abundant target proteins remaining in the resin. The eluents were collected (only six fractions) according to the original purification manual and were directly analyzed. Further characterization of the purified L1-HPV52 did not use the elution process since it lacks protein stability.

**Table 2:** Quantification of total protein concentration of recombinant L1-HPV52

Samples	Concentration (ug/mL)
Unpurified L1HPV52	5053.65
Eluent 1	39.33
Eluent2	38.37
Eluent 3	40.28
Eluent 4	34.56
Eluent 5	34.56
Eluent 6	67.96
Cleaved 1	1866.50
Cleaved 2	1154.0
Cleaved 3	772.50
Cleaved 4	697.70

**Fig. 7:**
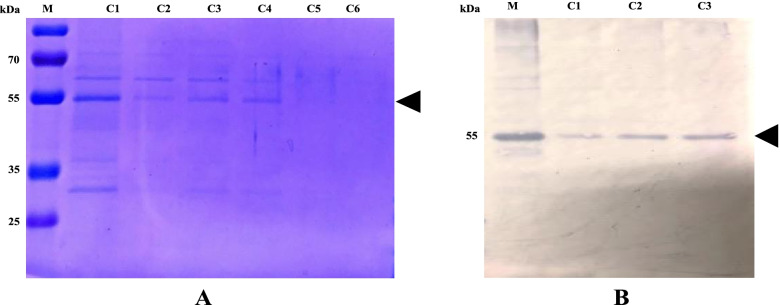
Proteolytic removal analysis of poly histidine-tagged SUMO fusion protein from L1-HPV52 **A** SDS-PAGE, **B** western blot with anti-L152-AP. Lane C1-C4, fraction of cleaved products; lane M molecular weight size marker (kDa)

**Fig. 8:**
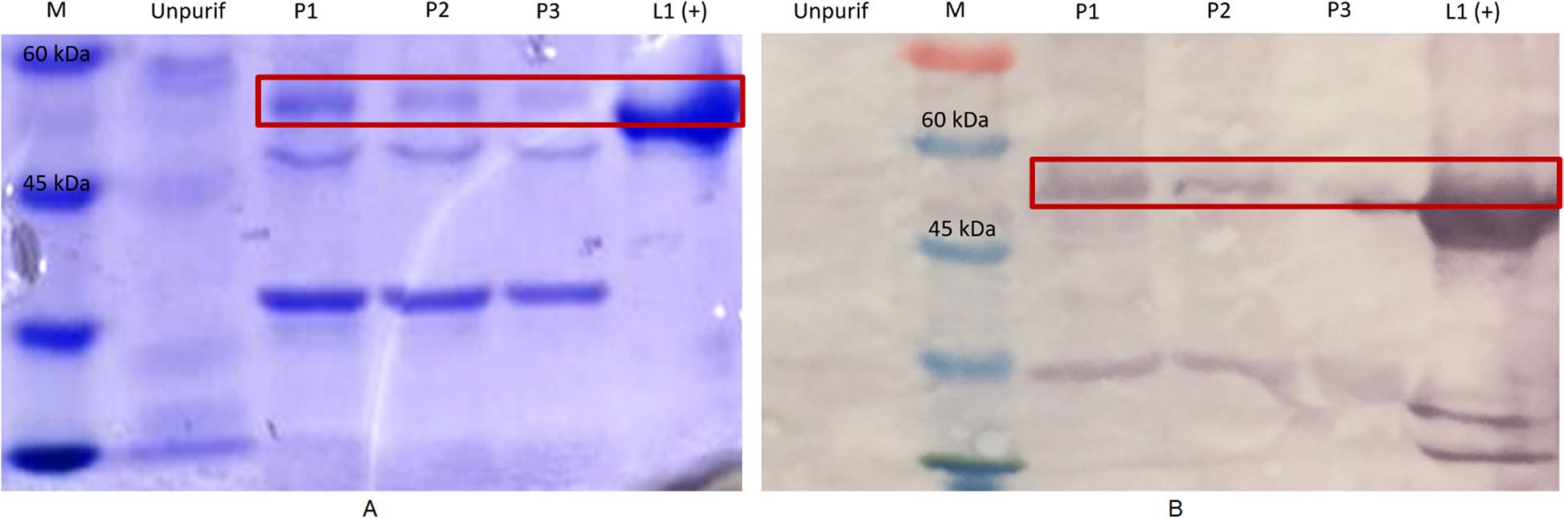
Proteolytic removal analysis of poly histidine-tagged SUMO fusion protein from L1-HPV52, compared to commercial L1-HPV52 **A** SDS-PAGE, **B** western blot with anti-L152-AP. Lane P1-P3 represent are fractions of our cleaved products; P4 is commercial L152 (Creative Diagnostic-DAGF-234) as a positive control, and lane M is molecular weight size marker (kDa)

To produce pure L1-HPV52 protein, the poly-histidine-tagged SUMO fusion of the purified L1-HPV52 recombinant was removed with SUMO protease. The SDS-PAGE analysis shows a clear band in the size of 55 kDa**,** and also in the immunoblot assay for the fraction cleaved products 1–4, and 5–6 had not detected any band. This indicates that there was a lack of the cleaved products (Fig. 7). More precise validation of the L1-HPV52 protein was also done, we made a comparison with commercial L152 (Creative Diagnostic-DAGF-234) (Fig. 8), and results show that our protein generates the same characteristic/pattern through immunoblot assay.

### Virus-like particles assembly of L1-HPV52 protein

**Fig. 9:**
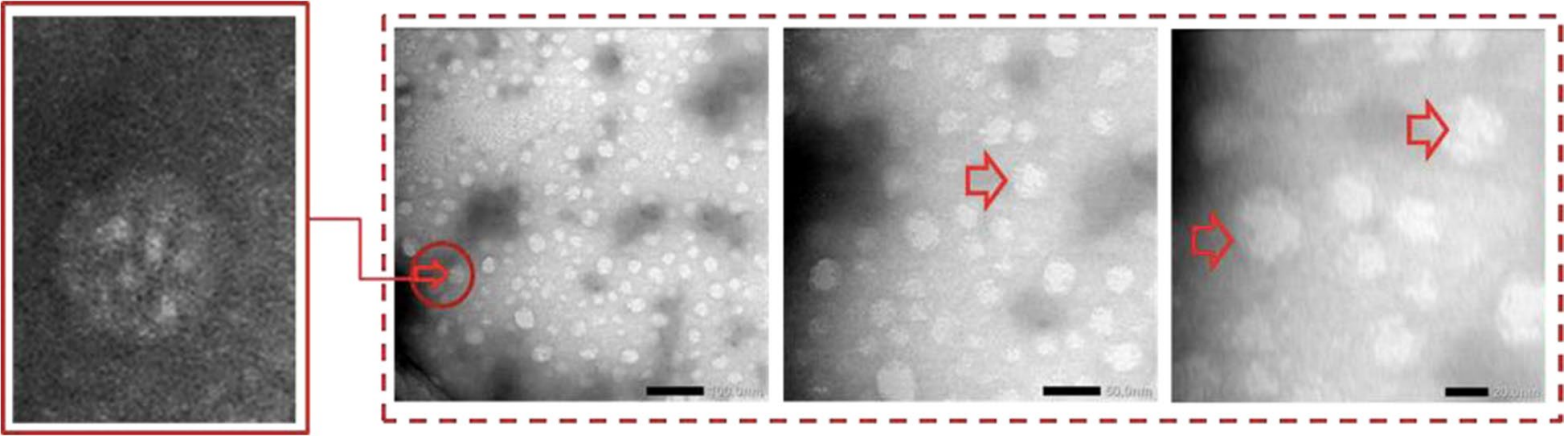
The TEM examination of assembled particles of L1-HPV52. The protein was incubated in the assembled buffer (pH 5.4) at 25°C for 30 min. Scale bars: 20–200 nm

In vitro assembly of VLP was conducted under defined and controllable conditions. The soluble form of HPV capsid protein is normally favorable for assembly. The L1-HPV52 without any fusion-tag protein had been successfully purified, well-characterized, and assembled in acid condition (pH 5.4). The transmission electron image of L1-VLPs was found to be homogenous in size, being 30–40 nm in diameter, and gave the mean at 26 nm (Fig. 9).

### Bioinformatic studies, assessment of B cell epitope prediction, T cell epitope prediction, and immunogenicity analysis

**Fig. 10:**
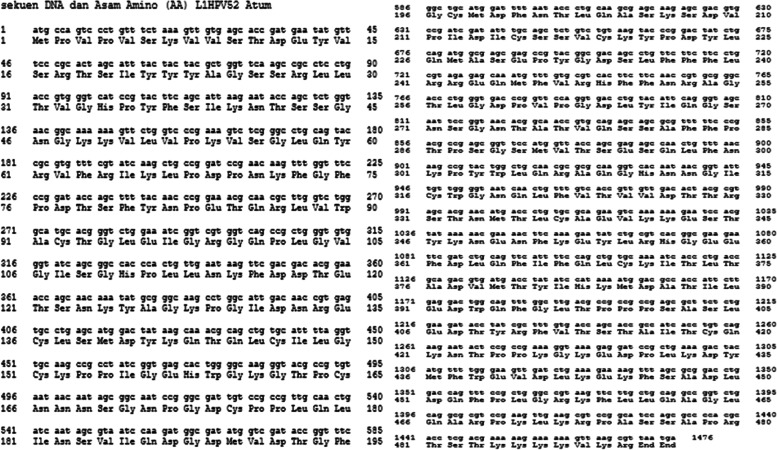
DNA and aa sequence as the result of sequencing and translated by Bioedit v7.2 and ExPASy v4.1

**Fig. 11:**
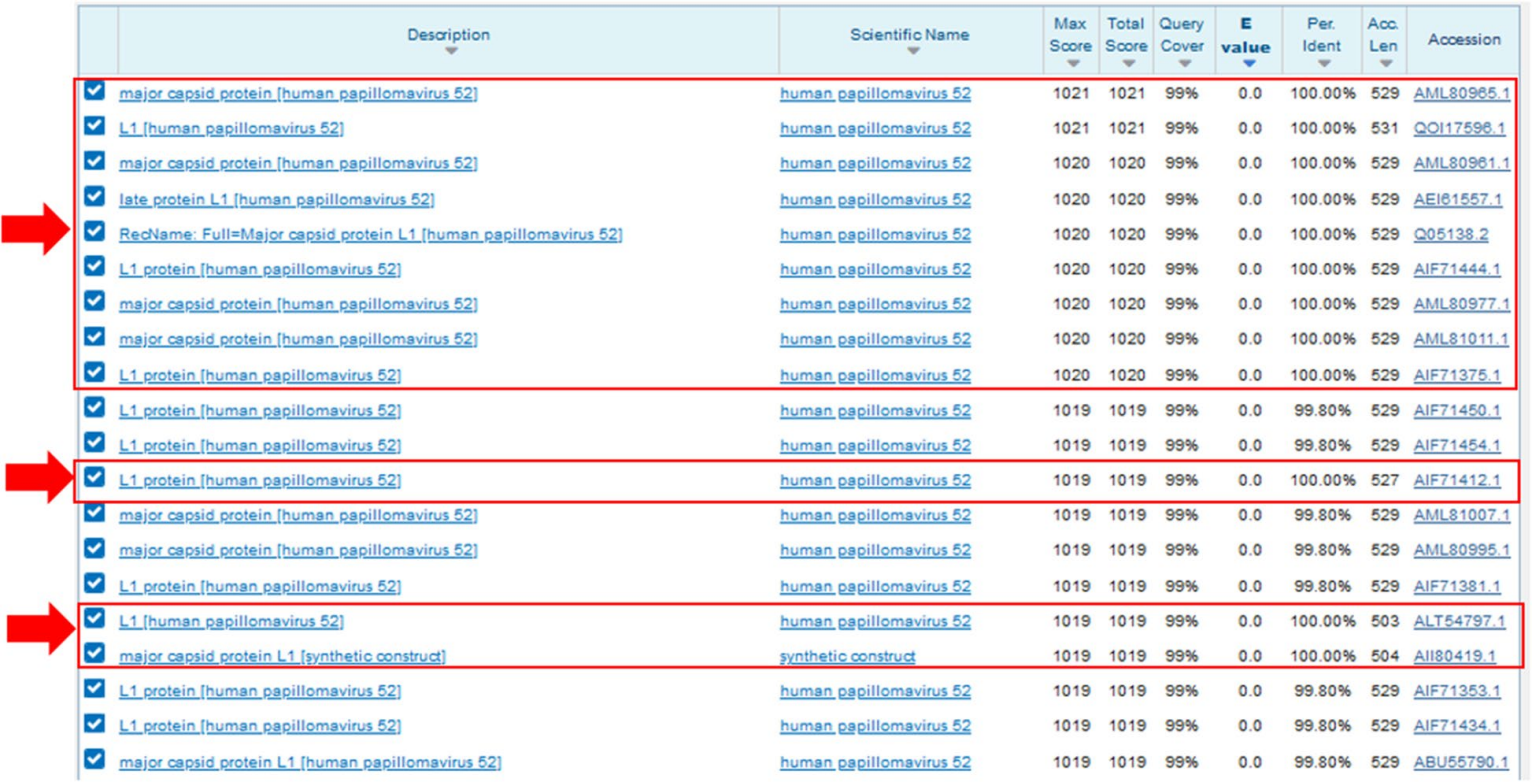
The BLAST analysis for aa L1HPV52 sequence

The bioinformatic study results can be observed at Figs. 10 and 11. Figure 10 shows the DNA and aa sequences of L1HPV52 that was cloned and expressed by using pD451-MR_L1 and pETSUMO in *E. coli* BL21 DE3. Figure 11 shows the BLASTp analysis results in aa level. The BLASTn for DNA level does not show.

From a total of 100 BLASTp sequences, only 12 sequences showed 100% identical. The sequences were from China (AML80965.1; QOI17596.1; AML80961.1; AML80977.1; AML81011.1; AII80419.1), USA (AEI61557.1), South Korea (AIF71444.1), Hong Kong (AIF71375.1), Italy (AIF71412.1), Brazil (ALT54797.1), and 3D structure of L1HPV52 pentamer in Protein Data Bank (PDB) coded Q05138.2.

Some B-cell epitopes prediction by using IEDB tools is shown on Table 3. There are 17 candidates. The average, minimum and maximum values ​of those 17 epitopes are 0.487, 0.218, and 0.710, respectively. Some epitopes (number 1, 2, 3, 10, 12, and 16) are conserved region (no mutation) when it was compared and aligned to other peptides in L1HPV52 from NCBI.

**Table 3:** Some B cell epitopes prediction.

No	Start	End	Peptide	Length	
1	6	15	SKVVSTDEYV	10	No mutation
2	40	49	KNTSSGNGKK	10	No mutation
3	74	85	GFPDTSFYNPET	12	No mutation
4	113	134	LNKFDDTETSNKYAGKPGIDNR	22	
5	155	173	IGEHWGKGTPCNNNSGNPG	19	
6	185	186	IQ	2	
7	190	190	M	1	
8	202	210	TLQASKSDV	9	
9	220	220	K	1	
10	223	232	DYLQMASEPY	10	No mutation
11	254	278	AGTLGDPVPGDLYIQGSNSGNTATV	25	
12	291	300	MVTSESQLFN	10	No mutation
13	341	360	KKESTYKNENFKEYLRHGEE	20	
14	387	413	ATILEDWQFGLTPPPSASLEDTYRFVT	27	
15	417	435	ITCQKNTPPKGKEDPLKDY	19	
16	443	454	KEKFSADLDQFP	12	No Mutation
17	464	488	GLQARPKLKRPASSAPRTSTKKKKV	25	

**Fig. 12:**
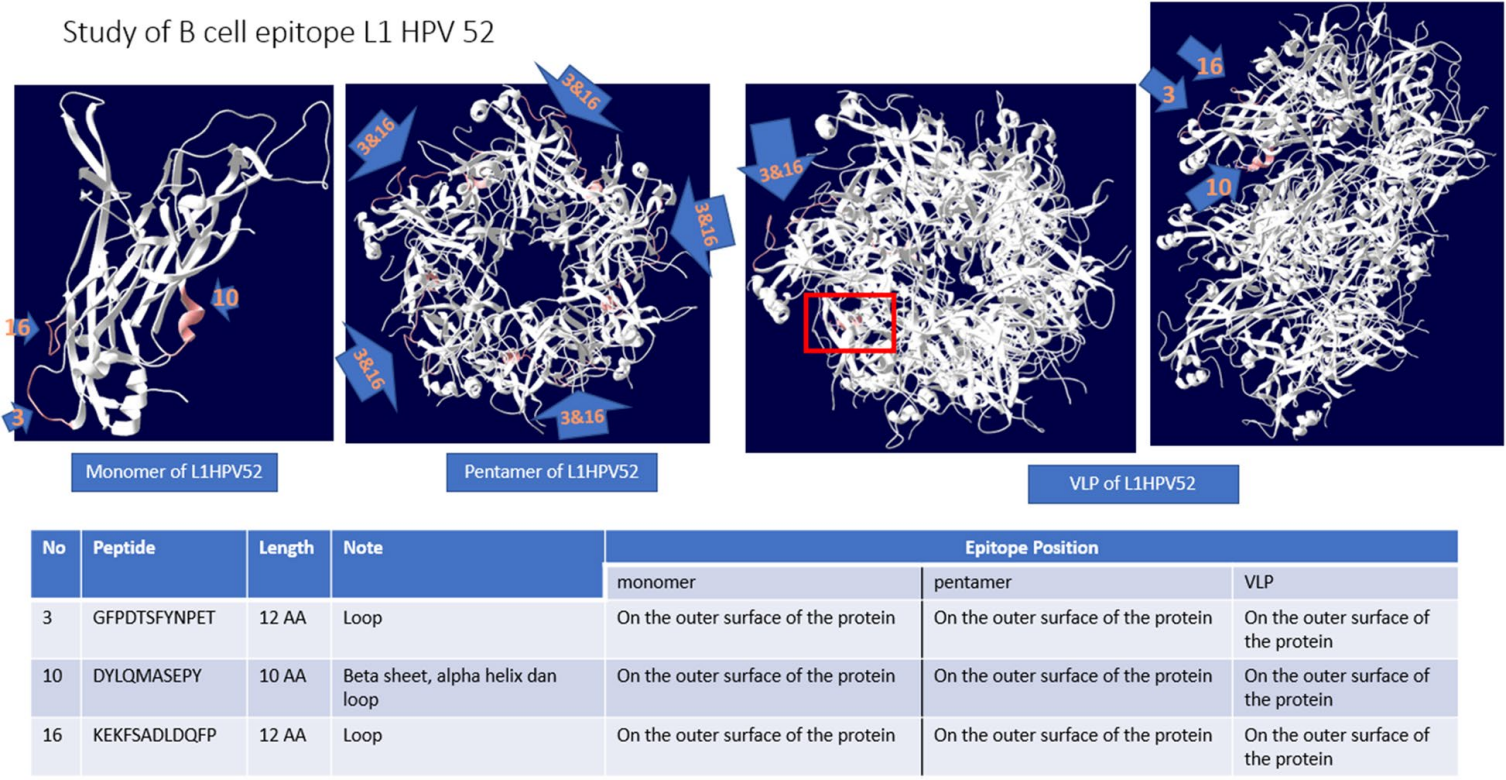
Prediction study of B cell epitope

Out of those 17 B cell epitopes sequences, there are only 3 epitopes that have no mutation, they are not too short or too long, and positioned on the outer surface of L1HPV52 protein in monomer, pentamer, and VLP forms. The position of each B cell epitopes was shown at Fig. 12.

**Fig. 13:**
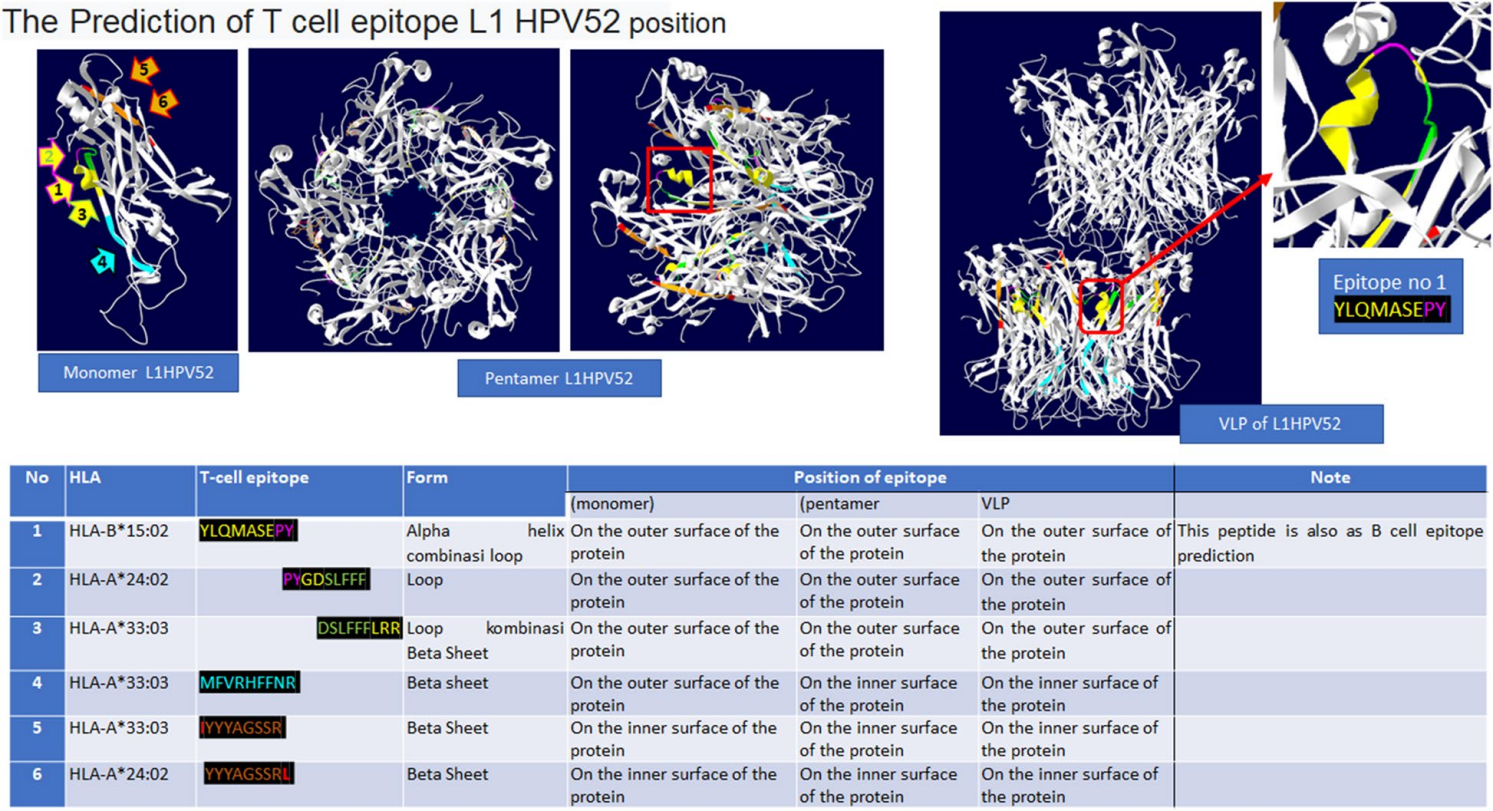
Prediction study of T cell epitope with class I of Indonesian HLA

The T cell epitope prediction study shows that there are six T cell epitopes that can attach to Indonesian HLAs class I (Fig. 13). Epitope no 1 can attach HLA-B*15:02. Epitope No. 2 and 6 can attach with HLA-A*24:02. Epitope no 3, 4, and 5 can attach with HLA-A*33:03. Furthermore, the number 1 Tm cell epitope is also predicted as B cell epitope (No. 10 on Table 3).

**Fig. 14:**
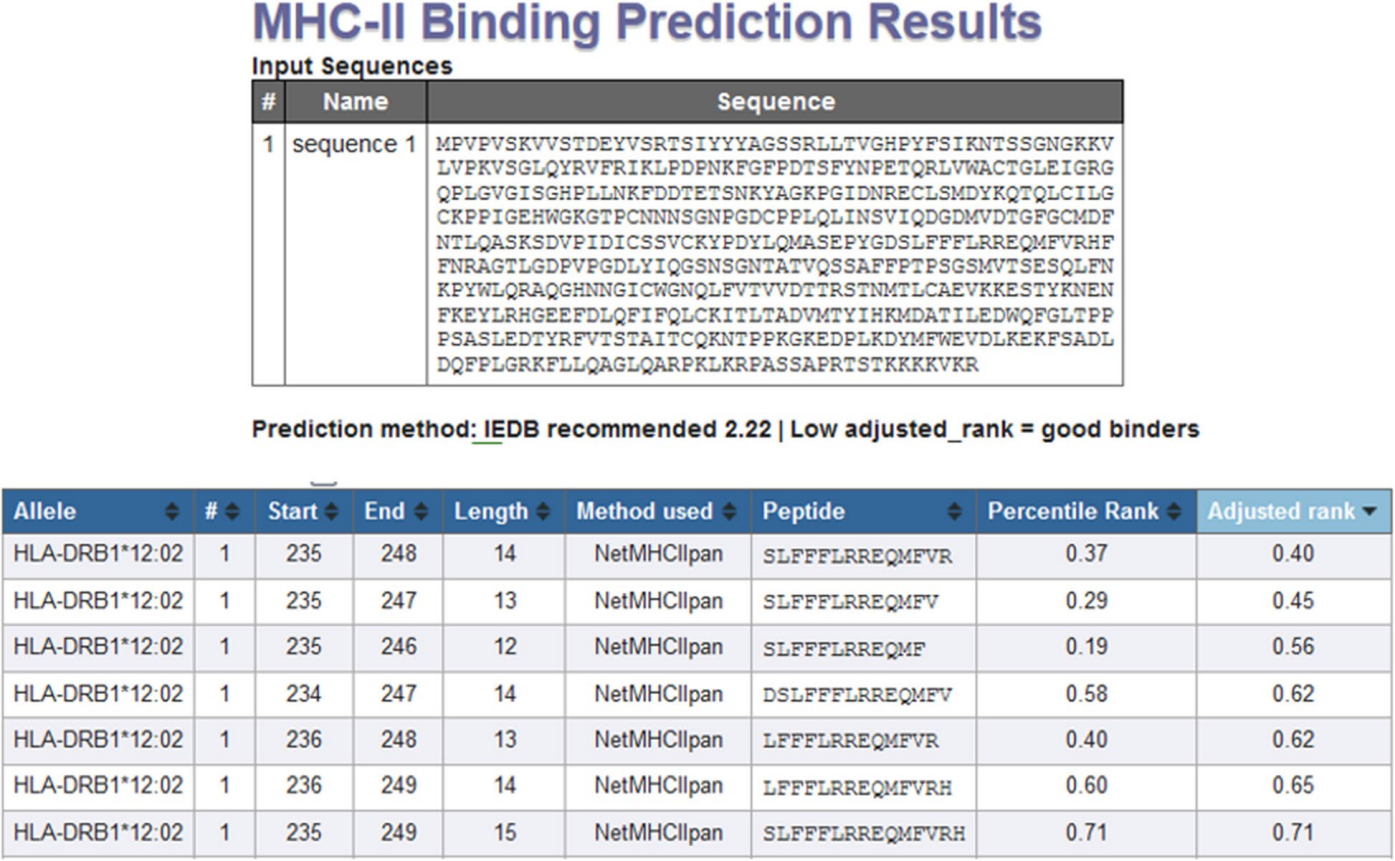
Epitope T cell Prediction with Indonesian HLA class II

The study about T-cel epitope prediction, which is focused on its binding to Indonesian HLA class II (HLA-DRB1*12:02), found T-cell epitope on aa from 234 until 249 (Fig. 14).

**Fig. 15:**
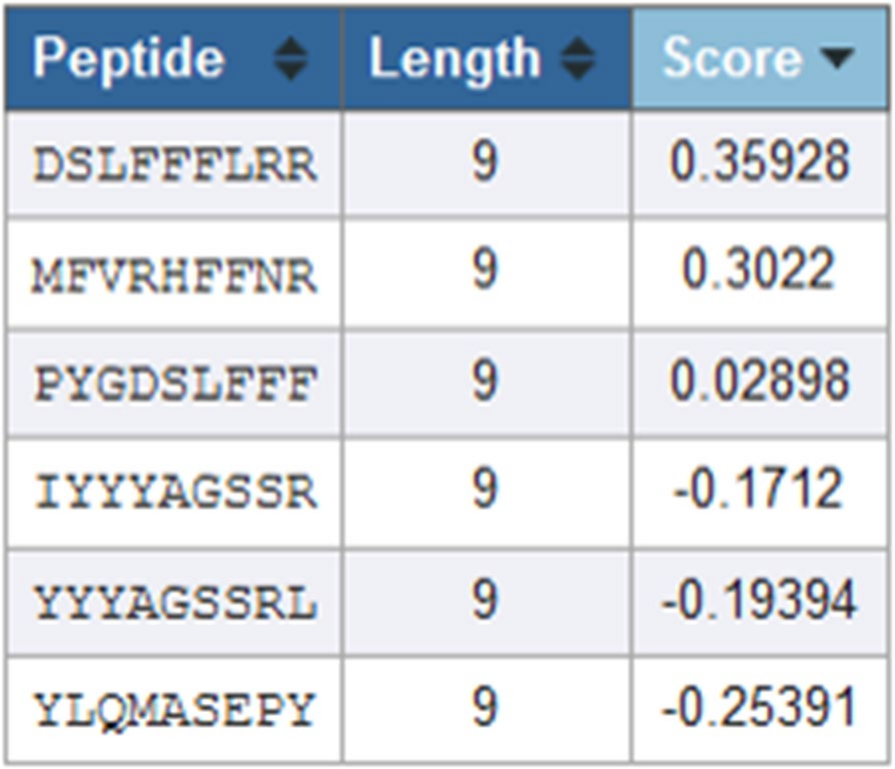
Immunogenicity of six T cell epitopes

Figure 15 shows that peptide DSLFFFLRR (marked as no 3 in Fig. 13) has the highest affinity value than others. A higher score indicates a greater likelihood of eliciting an immune.

## Discussion

Several strategic steps had already been reported in overcoming HPV cancers, and also, diverse difficulties need to be solved for a successful L1 protein expression, in order to meet the protein demands. The expression of L1 in *E. coli* was reportedly low, forming inclusion bodies that induce misfolded protein. Evidences suggest the truncation of the N-terminal and exclude the strong secondary structure inhibitor elements [17]. A recombinant construct of L1-HPV52 protein with 26 aa deletion of the N-terminal was developed, using an advanced pETSUMO expression system. Cloning of L1-HPV52 into appropriate vector is crucial for more efficient protein production that generates a high yield. The pETSUMO expression system employs TA cloning method that assures fast and efficient function [19]. Despite the challenges of using a bacterial expression system that generates inclusion bodies, pETSUMO solves the problem by enhancing the solubility of the partially insoluble protein. The SUMO tag became covalently conjugated to other proteins via an amide linkage, between C-terminal carboxyl and amino group in a lysine side chain [20]. Additionally, the expression system enabled the production of native protein by eliminating the poly histidine-tag SUMO fusion protein, which in turn, potentially affects the native conformation of the target protein [21].

This study showed that the recombinant protein with fusion tag remained in the resin when elution was done by 250 mM imidazole. It was hypothesized that poly histidine tag promotes oligomerization of the recombinant protein, which indicates higher imidazole concentration is needed to elute the protein [7]. Since the washing process is composed a low imidazole, the steps should be optimized to maintain the target protein. On the other hand, buffers play a significant role to maintain protein stability, where most of the protein stability was corroborated above the isoelectric point (pI). The isoelectric point of His-SUMO-L1HPV52 protein was 6.8, and the elution buffer used during the purification step was pH 8.0. The stability of the purified protein in this buffer did not persist for a long time because of the protein degradation process; therefore, the antibody was unable to detect any trace of purified protein through western blot analysis (data not shown). The occurred degradation process was caused by high concentrations of imidazole component in the elution buffer. The role of imidazole in protein degradation was explained as a catalytic reaction on histidine residues, therefore, purified protein with His-tag fusion was recommended to undergo dialysis with a buffer that maintains protein stability during storage [14].

In addition, some proteins with His-tag fusion are least stable in solutions for having pH values close to or lower than the calculated amount [22]. To overcome this challenge, the cleaved recombinant was directly processed further while it was in the resin, without eluting the protein and this decision successfully generated a soluble protein with single band at 55 kDa, which then was considered as an optimal procedure, to obtain the highest yield and concentration. Furthermore, the cleaved product was also comparable with the commercial L1 that was confirmed by immunoblot analysis. The SUMO fusion prevents protein aggregation even after the cleavage, due to its chaperone-like function to assist proper folding. Growth condition at 30 °C is good for expression of many SUMO fusion proteins, however, whenever the protein target is found insoluble at this temperature, it is then necessary to explore lower degrees (down to 15°C) [15]. In correlation to those previous findings, our results show similar outcomes, where the expression was performed at 20°C, it generated good results before and after the fusion cleavage.

In addition, to maintain the soluble native protein after cleavage and assembly into VLP, the L1-HPV52 was incubated in the low pH condition. The pH plays an important role for in vitro assembly, since it affects capsid-protein charge. Additionally, low temperatures are normally favorable as they reduce protein aggregation and chemical degradation. The L1 VLPs composed of 72-pentamers. Former research on HPV L1 has found that deletion of ten N-terminal residues led to assembly of a 12-pentamers rather than 72 [17]. Another research discoveries on Norovirus-like particles state that deletion of 34 and 98 amino acids of GII.4 Sydney (VP1) VLPs did not show any detectable particle with electron microscopy, however deletion of 26 and 38 amino acids introduced VLPs assembly [18]. This study is more likely to support the previous finding, where 26 amino acids truncated L1 protein successfully assembled into VLPs.

It is known that the size and homogeneity of observed particles depend on N-terminal truncation [15]. In our study, purified L1 VLP HPV52 showed variable particle size with mean ~26 nm, while the final yield of VLPs obtained was ± 6 mg/L. The heterogeneous sizes of the HPV L1 VLPs among different types were caused by a varied amino acid sequence in the N-terminal domains. Evidence suggests that the first 129 nucleotides in the 5′-end are composed of a strong RNA inhibitory component, and at least 10 and 30 residues were deleted from the N and C-terminus [17]. Truncation of ten residues in the N-terminal generated small L1 11/16 VLP with ~30 nm diameter [23], while 15 amino acid truncation generated L152 VLP with ~ 55 nm diameter [24].

The bioinformatic study out of sequencing result for L1HPV52 gene that has been inserted into pETSUMO has a total length of 1476 bp with 2 stop codons (TAA and TGA) on downstream of the gene. It expresses the major capsid protein (L1) HPV52 with a sequence length of 490 aa. Prediction of molecular weight, using bioedit v7.2, is 54846.63 Daltons (Da) or 54.9 kDa. The size is smaller than the native L1HPV52 because as many as 117 bp (39 aa) in the upstream of the gene were removed. The purpose of this partial deletion is based on a research conducted by Wei M and colleagues in 2018 [24], where they found that removal of 15 aa in the N-terminal of L1 HPV52 can increase their soluble expression in *E. coli* and in vitro self-assembly.

The B cells have an important role in HPV-associated cancer immunotherapy and response to cervical epithelial neoplasms and invasive cancers caused by HPV [26]. The EIDB results show that there are 17 B cell epitopes of L1HPV52. The sizes are varied, from 1 until 25 aa. Among those 17 candidates, 5 epitopes were selected, namely numbers 1, 2, 10, 12, and 16, based on their aa lengths that are neither too long nor too short. Other related studies of B cell epitope mentions that selection of a specific aa is usually made if it has not too long and short sequences, such as epitope studies on HPV 16 [27] and HPV33 and 58 [28]. In addition, this epitope has no mutations (conserved region) when it was aligned to 80 full coding sequences of L1HPV52 sin NCBI Genbank (Data not shown).

From these 5 epitopes, the 3 of them are located on the outer side of L1HPV52 protein, in all forms of L1HPV52 (monomer, pentamer, and VLP). These outer B cell epitopes were chosen regarding the fact that B cell only can recognize the outer epitope of an antigen. Attracting the B cells is important due to its function during the phagocytosis process when there is an antigen enters the body. If these three epitopes (number 3, 10, and 16) are recognized by B cell, then this B cell will engulf and degrade/break L1HPV52 antigen into smaller parts of peptides. Antigen phagocytosis by B cells is required for a potent humoral response [29].

The T cell epitope is associated with human leukocyte antigen (HLA). The HLA class I regions (HLA-A, B, and C) are carrying the highly polymorphic gene and those unique characteristic makes HLA precisely fit within its interaction through immunology view. The HLA class II regions (DP, DN, DM, DO, DQ, DR) are involved in antigen processing and presentation. While, the class III regions, contain genes that are implicated in inflammatory responses, leucocyte maturation, and the complement cascade [30].

The HLA recognizes foreign proteins (peptides) present in germs that enter the human body. If there is an interaction between HLA and peptide, the interaction formed will be brought to the cell surface and then recognized by T cells which will cause an immune reaction. HLA is highly selective and only binds to specific peptides, so it is important to predict the match between HLA protein and antigen peptide or T cell epitope so that their formation can trigger an immune response [31].

There are 6 T cell epitopes as a result of EIDB analysis of the L1HPV52 antigen. The T cell epitopes are YLQMASEPY, PYGDSLFFF, DSLFFFLRR, MFVRHFFNRA, IYYYAGSSR, and YYYAGSSSRL. These six epitopes were predicted can recognize several Indonesian HLAs class I (HLA-A*24:02, HLA-A*33:03, HLA-B*15:02). Observation on position of the T cell epitopes, especially on 3D structure of L1HPV52, some epitopes (number 1, 2, and 3) are located at/on the surface in both monomer and pentamer forms. Epitope number 4 is only on the surface when in monomer form. Meanwhile, epitope numbers 5 and 6 are located inside of the protein in form of monomers, pentamers, and VLPs. The epitope that can bind to Indonesian HLA class II (HLA-DRB1*12:02) is DSLFFFLRREQMFVRH.

The Indonesian HLA has similarities with HLA from South East Asia, especially for Java, Maluku, and Nusa Tenggara, namely HLA-B*15:02 and DRB1*12:02 (high frequency in Yogya). In addition, HLA class I related to Indonesians are A*24:07 (21.52%), A*33:03 (15.6%), A*24:02 (14.35%). While for Indonesian HLA class II were B*15:13 (11.18) and B*15:02 (11.6%) [32] [33].

## Conclusion

In conclusion, truncated gene encoding L1 HPV type 52 was successfully constructed, expressed, and assembled by *in vitro* method in acidic condition (pH 5.4), generating homogenous L1-VLP 30-40 nm size in the prokaryotic system of *Escherichia coli* BL21 DE3. Therefore, it is suggested to be promising and suitable for the development of L1 HPV type 52 vaccine.

## Data Availability

All data generated or analyzed during this activity are included in this published article.

## Abbreviations

HPV: Human papillomavirus
VLP: Virus-like particles
SDS-PAGE: Sodium dodecyl sulfate-polyacrylamide gel electrophoresis
LB: Luria Bertani
PMSF: Phenylmethylsulfonyl fluoride
IPTG: Isopropyl β-D-thiogalactoside
PCR: Polymerase chain reaction
TEM: Transmission electron microscope
BCA: Bicinchoninic acid
BSA: Bovine serum albumin
aa: Amino acid
HLA: Human leukocyte antigen
